# Getting an outsider’s perspective - sick-listed workers’ experiences with early follow-up sessions in the return to work process: a qualitative interview study

**DOI:** 10.1186/s12913-024-11007-x

**Published:** 2024-05-09

**Authors:** Martin Inge Standal, Vegard Stolsmo Foldal, Lene Aasdahl, Egil A. Fors, Marit Solbjør

**Affiliations:** 1 Faculty of Medicine and Health Sciences, Department of Public Health and Nursing, Norwegian University of Science and Technology (NTNU), Trondheim, Norway; 2 NTNU Social Research, Trondheim, Norway; 3 Unicare Helsefort Rehabilitation Centre, Rissa, Norway

**Keywords:** Return to work, Sick leave, Early follow-up, Interview study, Social insurance

## Abstract

**Purpose:**

The aim of this study was to explore how early follow-up sessions (after 14 and 16 weeks of sick leave) with social insurance caseworkers was experienced by sick-listed workers, and how these sessions influenced their return-to-work process.

**Methods:**

A qualitative interview study with sick-listed workers who completed two early follow-up sessions with caseworkers from the Norwegian Labor and Welfare Administration (NAV). Twenty-six individuals aged 30 to 60 years with a sick leave status of 50–100% participated in semi-structured interviews. The data was analyzed with thematic analysis.

**Results:**

Participants’ experiences of the early follow-up sessions could be categorized into three themes: (1) Getting an outsider’s perspective, (2) enhanced understanding of the framework for long term sick-leave, and (3) the empathic and personal face of the social insurance system. Meeting a caseworker enabled an outsider perspective that promoted critical reflection and calibration of their thoughts. This was experienced as a useful addition to the support many received from their informal network, such as friends, family, and co-workers. The meetings also enabled a greater understanding of their rights and duties, possibilities, and limitations regarding welfare benefits, while also displaying an unexpected empathic and understanding perspective from those working in the social insurance system.

**Conclusion:**

For sick-listed individuals, receiving an early follow-up session from social insurance caseworkers was a positive experience that enhanced their understanding of their situation, and promoted reflection towards RTW. Thus, from the perspective of the sick-listed workers, early sessions with social insurance caseworkers could be a useful addition to the overall sickness absence follow-up.

**Supplementary Information:**

The online version contains supplementary material available at 10.1186/s12913-024-11007-x.

## Introduction

Returning to work (RTW) from long-term sick leave is a complex and multifaceted process [1]. Prolonged sick leave has been linked to poorer health [2] and is thought to increase the psychosocial obstacles for RTW [3]. Therefore, early RTW interventions have been suggested to be central to the RTW-process [3]. Long-term sickness absence is often understood as sick-leave beyond 4–8 weeks of work absence. Most workers return to work on their own within the first few months of absence [4] and interventions in the following weeks, can improve the likelihood of RTW for those remaining [5–8]. Furthermore, in the context of long-term sick leave, interventions contributing to earlier RTW can be highly cost-effective [9, 10].

In Norway, the responsibility of early sick-leave follow-up is shared between the general practitioner (GP), who certify sick leave and assess remaining work capabilities, and the employer who should make accommodations at the workplace to facilitate RTW [11]. The employer has the main responsibility to assist their employees back to work but many employers lack the resources to properly facilitate RTW [12], and GPs may not see RTW as one of their primary focuses [13]. Thus, the existing system for early RTW follow-up in Norway, which largely rely on the cooperation between employer and employee, may not be sufficient to promote RTW [14]. This means that more effort to promote RTW might be needed. For instance, in other legislative systems RTW coordinators that assist other stakeholders and facilitate the RTW process are frequently used [15, 16]. In Norway, there are no formal RTW coordinator roles, and the task of facilitating cooperation between stakeholders, such as the employer, healthcare services and the sick-listed, fall on social insurance caseworkers working in the Norwegian Labour and Welfare Administration (NAV). They have a counseling role in sickness absence follow-up by providing support for the employer and sick-listed worker, but they also act as a controller of eligibility for sickness benefits [17]. Ordinarily, there are few meeting points between the sick listed worker and their NAV caseworker, and most sick listed workers have their first meeting with NAV when they have been sick-listed for six months.

The impact of RTW coordinators is contested. A broad systematic review determined that RTW coordinators had little effect on RTW [18]. However, face-to-face meetings with RTW coordinators have also been shown to increase RTW rates [19]. Evidence from Norway suggest that meetings between NAV caseworkers, sick-listed individuals and other stakeholders at 26 weeks could be cost-beneficial for RTW [20]. Caseworkers reviewing possibilities and barriers to RTW has also been found to improve the caseworkers’ knowledge of the sick-listed’s situation and consequently improved RTW rates in the following months [21]. Social insurance caseworkers could thus be in a position to provide additional case-management and support in the earlier stages of sick leave. Researchers have also suggested that NAV should play a more active part in the earlier phases of long-term sick leave [22]. Similarly, caseworkers have also called for being involved earlier in the RTW process [23]. In their experience, the longer workers are on sick leave, the harder it is to facilitate RTW [14]. Moreover, sick-listed individuals in Norway also expect some form of NAV involvement in the early stage of long-term sick-leave [24].

In a recent study, sick-listed workers experienced that early follow-up sessions where NAV caseworkers used motivational interviewing helped normalize their situation and improved their beliefs in their RTW plan [25]. Given the extensive resources required to implement and adopt motivational interviewing in a social insurance setting [23], it is also useful to know how early additional follow-up sessions without a guided focus is experienced, and how they could fit within the standard follow-up for workers on long term sick-leave.

Thus, the aim of this study was to investigate how sick-listed workers experienced early additional follow-up sessions with NAV and how they experienced the influence of the sessions on their RTW process.

## Materials and methods

The present study was based on 26 semi-structured individual interviews with sick-listed workers participating in a randomized controlled trial (RCT). The aim of the RCT was to evaluate the effect of motivational interviewing as an instrument for caseworkers at NAV in facilitating RTW for sick-listed workers [26]. The early follow-up sessions, which this paper focuses on served as an active control group.

### The Norwegian welfare system and sickness absence follow-up

In Norway, employees are entitled to full wage benefits in the case of sickness absence, from the first day of absence to a maximum period of 52 weeks. Sick leave is in most cases certified by the individual’s general practitioner. During the first 16 days, the employer is responsible for the payment, while the rest is paid for by the National Insurance Scheme through NAV [27]. The employer must initiate a follow-up plan in cooperation with the employee before the end of the fourth week of sick leave and is responsible for arranging a meeting with the sick-listed worker within the seventh week of absence, including other stakeholders if relevant. If the employer facilitates work-related activities, the sick-listed worker is required to participate. NAV is responsible for arranging a meeting including the employer and the sick-listed worker at 26 weeks of sick leave. The attendance of the sick-listed worker’s GP is optional. However, the GP is obliged to attend if NAV deems it necessary for the coordination of the RTW process. This is the only obligatory meeting point between a sick listed worker and NAV. Additional meetings can also be held if one or more of the stakeholders find it necessary. Thus, the sick-listed worker may also ask for a meeting with NAV to coordinate a plan for RTW outside this schedule [27]. After 12 months of sick leave, it is possible to apply for the more long-term benefits, work assessment allowance and permanent disability pension.

### The early follow-up sessions

The early follow-up sessions for this study were in addition to ordinary follow-up and consisted of two counseling sessions held at 14 and 16 weeks of sick leave. The sessions, offered by a NAV caseworker, lasted a maximum of 60 min and were in addition to standard NAV follow-up. During the first session, the caseworker opted to map out the sick-listed worker’s work situation, their relationship to their employer, their RTW plan, treatment plans and work ability, in addition to informing the sick-listed worker about their rights and duties as sick-listed. The caseworkers also informed about possible RTW measures through NAV. The second session focused on following up on the topics discussed in the first session, as well as focusing on any changes in the sick-listed workers’ situation that might have occurred between the first and second session.

These sessions functioned as an active control group in the RCT and were designed to be similar to the motivational interviewing sessions provided in terms of dose and timing. Caseworkers providing the active control sessions were separate from those providing the motivational interviewing sessions and they received no formal motivational interviewing training. They were, however, recruited voluntarily to the study from the same NAV-office as those performing the motivational interviewing sessions. Caseworkers were not randomized to group in the RCT and thus joined knowing that they would provide early follow-up using their usual methods.

### Study population and recruitment

The study population consisted of sick-listed workers who were enrolled in the RCT. Eligible participants were sick listed workers aged 18–60 years old, living in central Norway, with any diagnoses. Their sick-leave status at the time of inclusion in the RCT were 50–100% for at least 8 weeks. Exclusion criteria were pregnancy-related sick-leave, unemployment, and being self-employed. To be eligible to participate in this interview study the sick-listed worker had to have been randomized to the active control group in the RCT and completed the early follow-up sessions. Eligible participants were identified by NAV and contact info was forwarded to the researchers. A member of the project group invited the participants to take part in the research interview by phone. A total of 40 individuals were invited to participate in the interview study, of which 14 did not answer, declined the invitation, or did not show up at the interview. Twenty-six individuals participated in the interviews, including 19 women and 7 men aged 31–61. Participants showed diversity in their self-reported reasons for being sick listed, with 11 having mental health disorders, 8 having musculoskeletal disorders, and 7 individuals reported other disorders.

### Data collection

We conducted semi-structured individual interviews which allowed the participants to provide in-depth descriptions of their experiences. Interviews were based on an interview guide with five main questions concerning their experiences during sick leave, the RTW process, experiences of the two follow-up sessions, and whether these sessions led to any changes during their RTW process. The interviews were conducted between November 2018 and September 2019 and were audio recorded and transcribed verbatim. The duration of the interviews ranged from 35 min to 65 min.

### Data analysis

For our data analysis, we used reflexive thematic analysis which is a method for identifying, analyzing, and reporting patterns within qualitative data [28]. Thematic analysis is a flexible approach which allows researchers to interpret the data through a six phased recursive process, moving back and forth between phases to build themes from codes. The first step of the analysis involved becoming familiar with the data [28] where transcripts of all interviews were read and re-read by authors VSF, MIS and MS to get an overall impression of the contents. Preliminary codes and patterns were identified, as a start of the coding process. The second step of the analysis was the coding process, where items of interest related to the aim were coded by author VSF. These codes were then used to create core categories for further development of initial themes [28]. The third step was combining the codes into initial themes, which is a data reducing process which allows interpretation from the researchers [28]. Initial themes were discussed among all authors. The fourth step was reviewing the generated themes and checking them against the coded data, in order to further expand or revise the developed themes [28]. When reviewing the generated themes against the coded data, the preliminary analysis indicated a tendency where participants who received good support and follow-up by their employer considered the early follow-up sessions by NAV as less useful than the participants who lacked support and follow-up by their employer. However, a coding of the interviews focusing on this aspect showed no clear tendency of favoring early follow-up sessions based on high or low employer support. Thus, the initial themes were further developed into the three main themes which will be presented below. All authors had several meetings to discuss, define and refine the final themes in order to tell a coherent and compelling story about the data [28].

### Ethics

All participants received written and oral information about the study and gave their written consent before the interview started. Participants were informed that participation was voluntary and that they could withdraw from the study at any time, if the data had not been anonymized and integrated in the analysis.

The study was approved by the Regional Committee for Medical and Health Research Ethics in Southeast Norway (No: 2016/2300).

## Results

Regarding receiving the two sessions, the participants had overall positive experiences with the content and timing of the first session. The second session, however, was frequently experienced as an unnecessary repetition of the first as much of the content was already covered. In the following we present our results of participants’ experience of the early follow-up sessions as three themes: (1) Getting an outsider’s perspective, (2) enhanced understanding of the framework for long term sick-leave, and (3) the empathic and personal faces of the social insurance system.

### Getting an outsider’s perspective

Participants describe the meetings with a NAV caseworker as a positive experience that also challenged their current view of their situation and their RTW process. Meeting a NAV caseworker was experienced as an arena where they received guidance from an individual who examined their situation through an outsider’s perspective. NAV caseworkers provided support and encouragement, but also asked critical questions regarding their situation and their plans for RTW.

*“… we talked primarily about my situation, and I felt like I was allowed to talk to someone unbiased, without you know, being limited in the conversation. And I felt like I could talk about those things important to me. […] it turned out to be a good dialogue where she pulled me further, and made me think about a couple of things” - Interview 3*.

The outside perspective was described as useful due to the participants’ context prior to the meeting, which was their everyday lives with friends, colleagues, family, GPs, and employers. This informal network was described as significant supporters during the sick leave and served an important role as confidants to whom the sick-listed worker could talk about their difficult or confusing situation. The formal support from the employer varied, where some experienced several supportive phone calls and meetings with the employer during their sick leave, while others had only had a single formal meeting. Having support from the employer was experienced as crucial for a good RTW process, and absence of support and a distant relationship to the employer led to a difficult RTW process with negative emotions and reduced belief in their RTW capabilities. Participants also experience that being able to talk freely with the employer could be difficult, and that they would be held accountable if confiding about difficulties in RTW. Thus, in contrast to the largely supportive informal network, and the restrained environment surrounding employer-support, meeting the NAV caseworkers provided a useful outside perspective. When describing the early sessions compared to their overall sick leave follow-up, participants described meeting NAV as a calibration of their thoughts and providing a new perspective compared to their other RTW supporters.

### Enhanced understanding of the framework for long term sick leave

An important element of the first meeting was receiving information about rights, obligations as sick-listed, and the frame for future economic benefits. Receiving information about potential future loss of income and the possibility of having disability benefits was novel and useful for the participants. For some, this information led to new reflections on how being long-term sick-listed would have financial consequences, thereby providing another push for returning to work. For one participant, information about possible future loss of income provoked a feeling of panic and challenged her sense of identity.

*“I remember that when he started talking about work assessment allowance, I panicked a bit. Because I couldn’t identify with that category. But at the same time, I thought, okay, it’s good information to have you know.” - Interview 2*.

Furthermore, the participants were happy with agenda of the first meeting where the NAV caseworkers focused on short-term, as well as long-term plans for RTW and gave personal feedback about participants’ RTW plan. Included in the short- and long-term focus was receiving information from NAV about available RTW measures and interventions. Whether the sick-listed workers were planning on a fast or slow paced RTW plan, they experienced that receiving support on their plans and ideas strengthened their beliefs in managing RTW. NAV caseworkers also presented different strategies relating to possible accommodations at work, such as adjusting workload, work tasks and working time. Information such as the possibility of adjusting their time spent at work and their sick-leave status enabled the sick-listed workers to reorient their perception towards returning to work.

*“… in a way I hadn’t thought so carefully about when it’s smart to return and in what percentage. Because when I got that deal with the GP where I was still 100% sick-listed but could regulate it myself within 20% it was the first step to beginning to test myself.” - Interview 10*.

Participants received individually tailored information regarding the possibility of flexibility in the time spent at work and the amount of work they produced (i.e., sick leave percentage does not reflect hours spent at work, only the amount of work one does). This was highlighted as new and important information that was experienced as a contribution towards RTW.

### The empathetic and personal face of the social insurance system

All study participants had taken part in two sessions with a caseworker from NAV. Prior to these sessions, NAV had been perceived as difficult to get in touch with and some feared that cooperation with NAV would be either difficult or absent. However, when meeting the NAV-caseworker, their fears were diminished and to their surprise, they were met by supportive, accommodating, and friendly caseworkers.

*“NAV got a face; a personal face and NAV was no longer the huge colossus. The anonymous colossus that no one understands that just spews rules you have to relate to, which can be very … I can react with fear, I get afraid. “Am I doing this right?” you know. Am I following all these rules that I do not understand? What happened when NAV suddenly became a person was that they were on my side. They helped me, and it was possible to talk to NAV. A nice person helped me instead of rules that try to hinder me that I have to follow.” – Interview 19*.

The early follow-up sessions were experienced as more relevant when comparing them with other follow-up with their employer or later meetings with other caseworkers from NAV.

*“I wished that the other later conversations and meetings [with NAV] was comprised of the same understanding and competence that this counselor had. So that is what I’m sitting here thinking, that this was a star example of how one should be met, you know.” – Interview 5*.

The positive experiences of the early follow-up session were due to the understanding atmosphere that was created by the caseworkers, who was perceived as genuinely interested in their situation, cooperative and jointly reflecting about their RTW plan. Caseworkers asked questions about aspects of the participants’ lives that could be related to their situation as a sick-listed worker, and they appeared attentive when listening. This led to the experience of being met as a whole person and contributed to the early follow-up sessions being experienced as an arena where they felt acknowledged and cared for.

*“So, I came to NAV in high spirits and was well received and excellently informed and had a great conversation, really. Felt like I was to a psychologist, but that may be what I needed, and a neutral third-party that I feel listens to me. […] that is good medicine I think - that someone listens to what I say.” – Interview 6*.

Although some of the topics were considered quite personal, the sick-listed workers mostly experienced a respectful and reassuring dialogue with the caseworker. This personal and accommodating approach was overall positive for the participants, where the caseworkers matched their personality and behavior quite well. For several participants, the early follow-up sessions were considered almost therapeutic:

*“You know, I experienced [the sessions] very positively. I met a counselor that displayed a lot of understanding and for me it was almost therapeutic to talk to her. I sat there and though wow, either something has happened to NAV or this person is hand-picked for me.” – Interview 5*.

On the other hand, talking about health-related topics such as psychological well-being while being sick-listed could be emotionally straining. Some considered this therapeutic approach to a session as out of place. When these participants experienced questions from the caseworker as too personal, they saw their caseworker as intrusive and prying into personal issues. Such situations emphasized caseworkers’ position as representative for the social insurance system with its function for control and surveillance.

## Discussion

The results from this study showed that the participants experienced early follow-up sessions by social insurance caseworkers as positive. They described the value of receiving an outside view of their situation and practical information about being on sick leave, while at the same time being met with a supportive and respectful demeanor. These aspects were described as promoting reflection on their situation and their thoughts on RTW. The second session was, however, frequently experienced as superfluous and a repetition of the first session. This can also be seen in the results, where participants to a large degree describe the benefits of simply meeting an understanding NAV caseworker who provide practical information and helps them reflect on their situation, which could be achieved through a single session.

The sick-listed workers who experienced good supportive contact in the current study considered this to be instrumental for their RTW process. Comparatively, some sick-listed workers experienced an absence of support and a distant relationship to their employer. Supportive contact with the employer and workplace has been found to be critical in preventing work disability [29, 30] and important for facilitating RTW for sick-listed workers [31]. The negative impact of lack of workplace support on RTW has also been demonstrated previously [29, 30, 32, 33]. In the present study, participants to a large degree experienced support from their surrounding network. However, the type of support received has been suggested to play a role, where validation and empathy-based support may promote coping behaviors that are beneficial for RTW, while solicitousness could be detrimental through encouraging illness behavior [34]. Thus, an outside view of the situation at an early stage of sick leave may be sensible. The present study show that regardless of the support from other stakeholders, getting a second opinion was an exceedingly positive experience which provided an avenue for reflection upon their current situation and their plans going forward. Openness in the dialogue with caseworkers has also been identified as relevant to experience a fair and acceptable sick leave process [35], and RTW-coordinators arguably are in a position to provide an unbiased perspective on RTW plans, independent of the other stakeholders [36].

One of the benefits experienced in the present study was a greater understanding of the framework of sick leave. Social insurance literacy relates to the sick-listed individual’s understanding of the social insurance system, how to act on the information obtained, and why decisions surrounding their situation are being made [36, 37]. As individuals rarely have thorough knowledge of the social insurance system prior to sick-listing, social insurance literacy is also concerned with how well the system enables them to understand the process [38]. Previous research has suggested that enhancing the workers’ understanding of the system could improve their feelings of legitimacy and fairness in the process [35], and the present study provides some insight into how RTW coordinators could be experienced as helpful in this regard. Participants also described the clear agenda, in which the RTW plan was discussed, as useful. Examining barriers and facilitators for RTW and creating and re-examining the RTW plan is considered crucial to facilitate the RTW process [36]. The RTW-coordinator has also previously been suggested to have an important role in ensuring joint understanding and communication surrounding expectations and the context of long-term sick leave [39]. Thus, findings suggest that providing information on the system while inviting the sick-listed workers to reflect on their situation was experienced positively and possibly increased their social insurance literacy. However, the results in this study could also partly be explained by the context. It is possible that by voluntarily enrolling caseworkers and sick-listed workers in a research trial, a more individualized atmosphere was created in contrast to a more standardized RTW-follow-up scheme.

Nonetheless, experiences of the participants in the present study were largely positive and participants experienced being met with respect and understanding. Müssener and colleagues [40] also concluded in their study that how sick-listed individuals are treated affects their self-confidence and their perception of their ability to RTW. They suggest that the structural prerequisites for the RTW professional, such as having a gatekeeper role compared to a supportive role, seems to impact their treatment of sick-listed people [40]. The potential of the RTW coordinator to establish a good and trustful relationship with emphasis on the sick-listed workers’ motivation and resources in the RTW process has also been found to be important for RTW [41–43]. The conflicting roles of social insurance officers, being both facilitators and authority of benefits could potentially hinder the development of this relationship [41]. As identified by Karlsson [36], interactions between social insurance caseworkers and clients were perceived as either supportive or mistrustful. In the present study, the results suggest that the NAV-caseworkers may have had a stronger focus on the facilitator role, rather than the role of being gatekeepers of benefits.

In a recent study we found that sick-listed workers’ experienced early follow-up sessions with NAV as a positive experience and that it increased their RTW self-efficacy, when the caseworkers used motivational interviewing [25]. In the current study, the sick-listed workers met with NAV caseworkers who were not using motivational interviewing but rather using their ordinary approach when assisting sick-listed individuals. However, the experiences of the participants were strikingly similar in these two studies. The caseworker and sick-listed worker engaged in cooperatively reflections about when and how to RTW, which the sick-listed workers experienced to be valuable support and feedback for their RTW process. There may be some parallels to research on clinical psychotherapy, where studies have shown that the method of therapy may not be as important as the characteristics of the therapist [44, 45]. For instance, having interpersonal skills that enable a therapeutic alliance in which one can effectively promote a course of action and create belief in change is considered vital [46]. Thus, being met by an emphatic and understanding caseworker may be beneficial, regardless of approach to the sessions. The present study supports the notion that having an early face-to-face meeting with a NAV caseworker can be a positive experience in the RTW-process for long-term sick-listed workers.

Whether positive experiences with the social insurance system translates into RTW-rates is still debatable. On the one hand, a recent systematic review on RTW coordinators’ impact on RTW found that work absence duration and intervention costs were reduced when sick-listed workers had face-to-face contact with a RTW coordinator [19]. On the other hand, previous research has discussed the lock-in effect of programs through the social insurance service, which may lead to longer periods on sick leave [47]. Similarly, regular contact with the social insurance office has been shown to have a negative effect on RTW-rates, which may indicate the risk of developing a ‘social insurance career’ [48]. In a previous study we found that sick-listed individuals also experienced that caseworkers frequently recommended a slower RTW pace than what was originally planned [25]. Furthermore, even though the experiences of early contact with NAV-caseworkers in the present study was positive, no impact on RTW outcomes could be identified in the trial results [49].

### Strengths and limitation

A strength of the current study was the use of semi-structured interviews. This allowed the participants to elaborate and describe their experience of the early follow-up sessions in relation to their RTW process. In order to explore and uncover different experiences and nuances of the early follow-up sessions, a broad exploratory approach was used with a heterogenous sample. All analytical steps and preliminary findings were discussed with members of the research group to strengthen the interpretations, and final results were validated by all authors. The study also has some limitations. First, caseworkers performing the sessions voluntarily submitted to take part in the RCT and to undertake the follow-up sessions. They received no motivational interviewing training but were recruited from the same offices that those in the motivational interviewing group. This means there could be selection where caseworkers who were more interested in early follow-up were more likely to take part. Furthermore, there could be a spillover effect in the office, where caseworkers receiving motivational interviewing training pass on their knowledge to others in the office. We do however believe the impact of the spillover effect was small as recruitment was from one of the largest NAV-offices in Norway, and our previous study show that extensive training in motivational interviewing was required to achieve beginning proficiency [23].

Some participants in the study may have failed to recall information and details from the early follow-up sessions, since the interviews were conducted several months (ranging from 1 to 6 months) after the intervention. Although none of the participants expressed any difficulties in the interviews, there is a risk that the sick-listed workers held back information if they feared there would be consequences for their benefits. The current study recruited participants from a RCT with a response rate of approximately 15%. From this sample, the current nested study had a response rate of 65%. This indicates a selection bias, where participants agreeing to participate have different characteristics than those declining. Such bias might reduce variety in the experiences of the early follow-up sessions.

## Conclusion

Sick-listed workers considered additional early sessions with social insurance caseworkers as a positive addition to ordinary RTW follow-up. Having these early face-to-face meeting with respectful and accommodating caseworkers that also asked critical questions about participants’ situation, provided sick-listed workers with an outside perspective that enabled them to reflect on their situation. This was experienced as a useful addition to their friends, family and colleagues who were largely supportive. Furthermore, the sessions provided the sick-listed workers with an arena for receiving practical information on the framework of sick-leave follow-up, such as rights, obligations, and possibilities in strategies for RTW. This enabled them to adjust their plan towards RTW. Finally, having individual face-to-face sessions also changed participants’ perceptions of NAV from a anonymous entity to emphatic and understanding individuals, who seemed genuinely interested in assisting them back to work. Thus, from the perspective of the sick-listed individuals, early additional follow-up sessions were experienced as exceedingly positive and would be welcomed in addition to standard follow-up.

### Electronic supplementary material

Below is the link to the electronic supplementary material.

Supplementary Material 1

## Data Availability

To protect the anonymity of the participants, the datasets generated and analyzed during the current study are not publicly available. Redacted versions are available from the corresponding author upon reasonable request.

## Abbreviations

GP: General practitioner
NAV: Norwegian Labor and Welfare Administration
RTW: Return to work
RCT: Randomized controlled trial
