# Supplementary material for: Getting an outsider’s perspective - sick-listed workers’ experiences with early follow-up sessions in the return to work process: a qualitative interview study
Source: BMC Health Serv Res. 2024 May 9;24:609. doi: 10.1186/s12913-024-11007-x (PMC11080128; doi:10.1186/s12913-024-11007-x)
Supplement: Supplementary file 1 — Supplementary Material 1 [file 12913_2024_11007_MOESM1_ESM.docx]

**Interview guide – Experiences with early follow-up**

Main questions (numbered) and sub-questions/prompts (lettered).

*Introduction to the project and background information about the participant:*

**Can you please tell me a little bit about yourself?** (E.g., age, type of job / work tasks. Reason for- and length of sick leave. Number of follow-up sessions with NAV)

*Main questions:*

1. **Can you tell me about your situation as sick listed and the process of returning to work?**
2. **Can you tell me about the type of follow-up you have received as sick-listed, and from whom? (E.g., general practitioner, employer, health services, social insurance)**
3. **Can you tell me about the two follow-up sessions with NAV?**
   1. What did you talk about in these sessions?
   2. How did you experience the relationship with your caseworker?
   3. Did you have any expectations going into the sessions?
   4. How did you experience the timing of the sessions relating to the length of sick leave you had?
4. **What consequences did the session have for you (if any)?**
   1. Work, family, economic, habits, other things?
   2. Did you reflect on anything after these sessions? (E.g., situations, relations, behavior)
   3. Did you make a plan after the sessions?
5. **What do you think about the future?**
   1. About returning to work?
   2. Why is returning to work important/unimportant for you?
   3. What expectations do you have for returning to work?
   4. Who supports you in your return to work process?
